# Intimate partner violence: psycho-physio-pathological sequelae for defining a holistic enriched treatment

**DOI:** 10.3389/fnbeh.2022.943081

**Published:** 2022-09-30

**Authors:** Valentina Cesari, Alessandra Vallefuoco, Jacopo Agrimi, Angelo Gemignani, Nazareno Paolocci, Danilo Menicucci

**Affiliations:** ^1^Department of Surgical, Medical and Molecular Pathology and Critical Care Medicine, University of Pisa, Pisa, Italy; ^2^Department of Biomedical Sciences, University of Padua, Padua, Italy; ^3^Clinical Psychology branch, Azienda Ospedaliero-Universitaria Pisana, Pisa, Italy; ^4^Division of Cardiology, Department of Medicine, The Johns Hopkins Medical Institutions, Baltimore, MD, United States; ^5^Comitato Unico di Garanzia, University of Pisa, Pisa, Italy

**Keywords:** IPV, environmental enrichment, psychotherapy, psychological support, safe environments, empowerment

## Abstract

Intimate partner violence (IPV) is a health priority, which worldwide, mainly affects women. The consequences of IPV include several psychophysiological effects. These range from altered levels of hormones and neurotrophins to difficulties in emotion regulation and cognitive impairment. Mounting evidence from preclinical studies has shown that environmental enrichment, a form of sensory-motor, cognitive, and social stimulation, can induce a wide range of neuroplastic processes in the brain which consistently improve recovery from a wide variety of somatic and psychiatric diseases. To support IPV survivors, it is essential to ensure a safe housing environment, which can serve as a foundation for environmental enrichment-based interventions. However, some concerns have been raised when supportive housing interventions focus on the economic aspects of survivors’ lives instead of the emotional ones. We thus propose a holistic intervention in which supportive housing is integrated with evidenced-based psychotherapies which could constitute an enriched therapeutic approach for IPV survivors.

## Introduction

Intimate partner violence (IPV) refers to physical/sexual violence, stalking, or psychological injury perpetrated by a current or former partner/spouse. It represents a public health priority since worldwide it affects 27% of women aged 15–49 over their lifetime with physical and mental impacts on the victims’ health (World Health Organization, 2013). Epidemiological research (Fanslow et al., 2022) surveying both women and men suggests that women are disproportionately victimized, with 12.4% of women and 2.1% of men reporting any lifetime IPV.

The lifetime prevalence of IPV perpetrated against women varies across regions with the highest prevalence rates reported in the Western Pacific (60.0%–68.0%), South East Asia (37.7%), Eastern Mediterranean (37.0%), and Africa (36.6%), compared to the Americas (29.8%), Europe (25.4%), and high-income regions (such as Australia, Canada, UK, etc.; 23.2%). Such variability in IPV rates across regional groups suggests that there are specific differences in IPV based on culture and ethnicity (World Health Organization, 2013; Miller and McCaw, 2019).

In IPV, physical violence includes aggressive behaviors and controlling access to physical necessities comprising sexual abuse and stalking. Sexual abuse comprises forced sex, coercive sex with a partner or other people, violence associated with sexual assault and rape, interfering behavior that impedes birth control measures, and protection against sexually transmitted infection. Stalking comprises a pattern of repeated, intrusive actions—such as following, harassing, and threatening—which cause fear and distress in victims and psychological injury including words and behaviors aimed at intimidating, degrading, humiliating, or isolating the victim (Marx et al., 2013).

The consequences of IPV include the disruption to victims’ lives, both socially and economically, chronic and debilitating physical and mental health problems and can even result in death (Campbell, 2002; Ellsberg et al., 2008).

In terms of psychopathological symptoms, IPV includes several psychological alterations, of which posttraumatic symptoms are the most common, along with a high number of comorbidities such as mood and anxiety disorders (Karakurt et al., 2014). Survivors’ experiences of psychological symptoms have been reported to be highly variable, and can be ascribable to personal strengths and resources, duration and severity of the abuses, experience of other lifetime traumas, and access to services and social support. A higher risk of developing PTSD has been reported with increasing types of abuse suffered (physical, psychological, or sexual; Pico-Alfonso et al., 2006; Fedovskiy et al., 2008).

During the COVID-19 pandemic, it has been reported an increase of IPV phenomena. According to data collected from Kenya, Thailand, Ukraine, Cameroon, Albania, Bangladesh, Colombia, Paraguay, Nigeria, Cote D’Ivoire, Morocco, Jordan, and Kyrgyzstan, since the COVID-19 pandemic, 45% of women reported that they had either undergone a form of violence or knew a woman who had been a victim (UN Women—Headquarters, 2022).

Due to the impact of IPV on victims and on relevant stakeholders (families, friends, healthcare system), the optimization of therapeutic approaches based on personalized survivors’ needs is mandatory. Herein, we propose a new therapeutic framework based on environmental enrichment (EE), a form of sensory-motor, cognitive, and social stimulation, inducing several neuroplastic processes in the brain to normalize psychopathological alterations, and improve survivors’ quality of life. For this purpose, we start discussing the biopsychological sequelae that can follow IPV exposure. Considering that EE can reverse most of them, enhancing current approaches based on EE-related concepts might help healthcare professionals to better manage IPV survivors, and provide a potential to improve survivors’ quality of life.

## Biopsychological Signs of IPV

IPV can be defined as a form of social (interpersonal) stressor, often systematic and chronic, due to the actions of the perpetrator toward survivors. Beyond the direct effects of violent actions, the coercive control exerted by perpetrators has an impact on the survivors’ life, limiting social contacts and access to professional help (Wilson, 2012, p. 60; Robinson et al., 2018).

Importantly, many IPV survivors experienced physical and emotional alterations as a consequence of being forced into isolation by controlling perpetrator (James et al., 2004). The impoverished environment (violence and social isolation) in which IPV survivors are forced to live can lead to widespread psychophysiological distress such as endocrine (Cacioppo et al., 2015) neurotrophic (Zaletel et al., 2017), psychopathological (Baek, 2014), cardiac and autonomic alterations (Grippo et al., 2007). In the following sections, a description of the aforementioned consequences, derived from an impoverished environment, is reported.

### IPV and endocrine correlates

**Figure 1:**
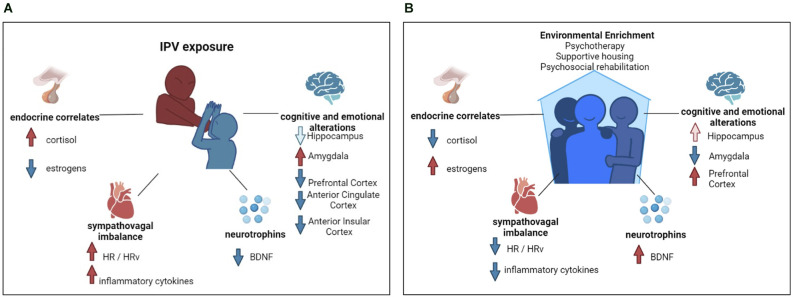
Flow chart of intimate partner violence’s psycho-physio-pathological sequelae and environmental enrichment beneficial effects. **(A)** Graphical representation of biopsychological sequela of intimate partner violence survivors. Red arrows indicate an increase; blue arrows indicate a decrease. In the graphical representation of cognitive and emotional alterations, the light blue arrow indicates brain structure volumetric decrease, whereas the red and the blue arrow indicate functional alterations. **(B)** Graphical representation of the biopsychological benefits of environmental enrichment. Red arrows indicate an increase; blue arrows indicate a decrease. In the graphical representation of cognitive and emotional alterations, the light red arrow indicates brain structure volumetric increase, whereas the red and the blue arrow indicate functional alterations.

From an endocrine standpoint, IPV survivors have shown an abnormal daily time course of cortisol levels, with a lower cortisol in the morning and a higher one in the evening or permanently elevated diurnal cortisol output in chronically stressed individuals (Miller et al., 2007; Bellagambi et al., 2018; Yim and Kofman, 2019) compared to healthy subjects (Figure 1A).

A higher morning salivary cortisol level has also been found in women with respect to men (Larsson et al., 2009) and thus might represent a vulnerability toward mental stressors (Dziurkowska and Wesolowski, 2021) such as IPV.

Another hormone intimately linked to stress exposure-response is estradiol (Figure 1A). In fact, the level of estradiol is higher when there is a lower level of perceived stress (Roney and Simmons, 2015). Fluctuations in estrogen levels may be related to difficulties in fear of extinction, thus contributing to post-traumatic symptom maintenance (Glover et al., 2015). The fact that women are the most vulnerable to the consequences of IPV could be attributed to the role of this hormone in female physiology.

However, as no studies have been conducted to differentiate IPV survivors with or without psychiatric diagnosis (e.g., post-traumatic symptoms) according to the circadian course of cortisol and estradiol; we cannot exclude that abnormal levels in IPV reflect the exacerbation of psychopathological symptoms instead of being a marker of exposure to violence.

### IPV and neurotrophins

The aforementioned fluctuations in cortisol and estrogen are interrelated with the level of brain-derived neurotrophic factor (BDNF), a neurotrophin involved in synaptic plasticity (Thoenen, 1995), and in the maintenance of midbrain dopaminergic (Hyman et al., 1991) and cholinergic neurons (Ha et al., 1999). Insufficient production or deficiency in the use of BDNF can lead to several dysfunctions (Connor and Dragunow, 1998).

Low levels of peripheral BDNF have been found in psychiatric disorders such as mood disorders, schizophrenia, suicidal attempts, and PTSD (Dell’Osso et al., 2009). Regarding traumatic exposure, evidence has been found of increased BDNF methylation in PTSD, however the results of studies exploring BDNF concentration levels in the blood of these patients are mixed. BDNF levels have been associated with the duration of depression symptoms only in women (de Azevedo Cardoso et al., 2014). Some studies have shown increased concentration levels of BDNF (Matsuoka et al., 2013; Wu et al., 2021), others have reported decreased concentration (Dell’Osso et al., 2009; Angelucci et al., 2014); however, all the studies have highlighted an essential function of BDNF in PTSD.

Despite the lack of studies focusing on IPV survivors, it could be argued that abnormalities in BDNF levels or circadian patterns might act as both predisposing and perpetuating factors in psychological sequelae (Figure 1A). Some animal models suggest a clear role of BDNF in mediating social stress-induced damage. Levels of BDNF and its specific receptor, tropomyosin kinase B, were found to markedly lower in the hippocampus and heart of obese mice exposed to a psychosocial stress paradigm (Agrimi et al., 2019). This role of BDNF is also confirmed by clinical models: lower BDNF plasma levels have been associated with childhood physical neglect in depressed women (Grassi-Oliveira et al., 2008), bipolar patients with a previous history of trauma (Kauer-Sant’Anna et al., 2007), victims of sexual abuse or accidental injury (Kauer-Sant’Anna et al., 2007), or in those suffering from burnout syndrome (Buselli et al., 2019).

### Cognitive and emotional alterations in IPV survivors

IPV acute- and long-term effects also negatively impact the cognitive domains of survivors, including attention, memory, and learning. Studies found a significant positive correlation between IPV history and long-term decay in cognitive performances (Williams et al., 2017) likely due to the strong involvement of cognitive functions in emotional processing. It has also been argued that this cognitive-emotional impairment might also preclude the IPV survivors’ response to treatments, thus exacerbating the psychopathological sequelae of IPV. Chronic abuse, to which IPV survivors are often exposed, might induce a pathoplastic mechanism with alteration in the brain regions sustaining cognitive performances: the hippocampus (particularly involved in memory processes), amygdala (fear and emotional regulation), anterior insular cortex (interoceptive attention), anterior cingulate cortex (ACC; cognitive influence on emotions), and prefrontal cortex (PFC; cognitive control; Bolsinger et al., 2018; Figure 1A).

For the limbic system structures, sex differences have been found in the amygdala resting state functional connectivity in relation to internalizing symptoms severity. This suggests that, for females, higher internalizing symptoms are associated with greater connectivity between the amygdala and regions involved in emotional and somatosensory processing, salience detection, and action selection (Padgaonkar et al., 2020).

The volume of the hippocampus and its relationship with neuroendocrine factors, such as the reductions of BDNF, impacts cognitive performances (von Bohlen Und Halbach and von Bohlen Und Halbach, 2018), and might be a neuroanatomic target for PTSD. The development of PTSD seems also to be related to a pre-existing smaller hippocampal volume, which interferes with the subjective ability to recover from a traumatic event (Gilbertson et al., 2002; Kremen et al., 2012). The association with a smaller hippocampal volume is primarily due to a strong negative association in females with PTSD (Logue et al., 2018). An overactivation of the parahippocampal gyrus (playing a crucial role in associative learning and in memory encoding and retrieval) in response to non-threatening stimuli, identified by studying event-related potentials (ERPs) such as N170 and late positive potential (LPP), has suggested that experiencing IPV during a lifetime can modulate emotional appraisal (Pointet et al., 2021).

The frontal cortex, along with the insula (primarily involved in interception), plays a crucial role in multiple cognitive functions and affects emotional processing and regulation in IPV survivors.

In fact, a reduction in gray matter revealed by magnetic resonance imaging (MRI) enabled patients with PTSD and a history of IPV in their lifetime to be distinguished from those with PTSD only (Fennema-Notestine et al., 2002).

The insula and the prefrontal cortex are key in emotional anticipation (Aupperle et al., 2012) and emotional processing. Women with IPV showed increased activation of the anterior insula and amygdala and decreased connectivity among the anterior insula, amygdala, and ACC during the match of fearful vs. happy target faces, and similarly also for angry vs. happy target faces. Also, IPV showed an increased dorsal ACC/medial prefrontal cortex activation and decreased ventral ACC activation when matching to a male vs. a female target (Fonzo et al., 2010).

This leads to specific behaviors common to IPV survivors, like an exaggerated response to contextual cues that relate to the IPV trauma.

The right dorsolateral PFC and middle insula show signs of increased activation also during painful stimulations. If subjected to repeated painful stimulations, there is decrease in the activity in these areas (Strigo et al., 2010). This suggests why numbing and avoidance are common in IPV survivors. PTSD as a consequence of IPV may relate to a decreased neural flexibility in tasks that require inhibition on a PFC level (Aupperle et al., 2016), which may affect the cognitive performances of survivors and induce specific behaviors that could prevent the survivors’ rehabilitation.

This complex framework of emotional and cognitive alterations resulted to be exacerbated by sleep disturbances, which often co-occur following IPV experiences (Maharaj et al., 2020). These sleep alterations are often linked to psychiatric disorders such as PTSD (Gallegos et al., 2021), and which might be fuelled by subjective maladaptive strategies such as metacognitive beliefs (Palagini et al., 2014). More generally, sleep disturbances, particularly insomnia, are associated with impaired cognitive performance (Wardle-Pinkston et al., 2019).

### IPV and sympathovagal imbalance: evidence from peripheral indices

IPV is associated with dysfunctions in the autonomic nervous system, with alteration in the sympathovagal balance (Schneider and Schwerdtfeger, 2020). For example, IPV survivors showed a higher cardiovascular reactivity (heart rate, vagal ratio, and pre-ejection period) when exposed to stressful tasks (O’Neil and Scovelle, 2018; Constantino et al., 2019), thus highlighting the cardio-toxic effect of the violence perpetration (Figure 1A). The study of inflammatory markers from saliva in IPV survivors suggests that changes in inflammatory pathways might be associated with, and could prompt cardiovascular risk as well as an autonomic imbalance. In fact, these two domains are tightly connected: the autonomic imbalance, and specifically sympathetic over reactivity, is associated with the release of certain pro-inflammatory markers (Figure 1A). In fact, the exposure of IPV survivors to chronic stress is associated with the sympathetic nervous system and hypothalamic-pituitary-adrenal axis which activates the release of cardiovascular disease biomarkers such as catecholamines, glucocorticoids, and inflammatory cytokines (De Bosscher et al., 2008; Nikkheslat et al., 2015). An increase in inflammatory markers, including C-reactive protein and interleukin-6 has also been found in IPV survivors (Newton et al., 2011).

Regarding the comorbidity of IPV and other psychiatric diseases, increased sympathetic tone, paralleled by a dramatic reduction in vagal tone, have been found. A higher heart rate and electrodermal activity have been detected in IPV survivors during exposure to trauma-related stimuli and resting-state monitoring. Women are thus more vulnerable to cardiovascular alterations due to their basal higher heart rate, and these sex differences in cardiovascular functioning may be exacerbated by trauma (Saltzman, 2021).

Lower respiratory sinus arrhythmia—the heart rate variability in synchrony with respiration—has been found at baseline and in response to trauma reminders (Blechert et al., 2007) in PTSD women with a history of IPV, thus indicating a reduced parasympathetic activity and a hyperarousal state (Hopper et al., 2006).

Lastly, women with a premenstrual dysphoric disorder who experienced abuse throughout their lifetime showed significantly higher blood pressure and vascular resistance index during the resting state (Girdler et al., 2007). All these alterations in the autonomic nervous system could explain the vulnerability to cardiovascular diseases of IPV survivors.

Besides the effect on the cardiovascular system, the higher autonomic activity might prompt an excessive and chronic alerting state, causing a neuro-hormonal imbalance, pro-inflammatory activity, and psychological sequelae such as PTSD, anxiety, and depression that might parallel cognitive impairment (Laurino et al., 2012). The negative effects on cognitive functions might cause IPV survivors to enter a vicious circle that could hinder survivors’ rehabilitation.

## Environmental Enrichment: Psychophysiological Effects

IPV survivors are exposed to social stressors, of which violence and social isolation are the most common. This extremely impoverished environment might exacerbate all the aforementioned psychophysiological alterations. To face this form of violence, a therapeutic intervention based on emotional and social needs to compensate for the lack of environmental positive stimulation during violence exposure is highly recommended. To this aim, the use of interventions based on environmental enrichment (EE) has the potential to be the most appropriate.

Although environmental enrichment is a multifaceted form of sensory-motor, cognitive, and social stimulation, leading to a wide range of neuroplastic processes in the brain, it is rarely used in clinical setting (Livingston-Thomas et al., 2016). EE enhances learning and memory abilities, promotes various forms of neuroplasticity, and improves recovery from a wide variety of somatic and psychiatric diseases. Perceived social support in an enriched environment is associated with mental health recovery from anxiety, depression, and PTSD (Blasco-Ros et al., 2010).

From a biological perspective, EE could benefit neuronal health, which is usually severely impaired after traumatic and repeated events, such as IPV (Figure 1B). EE positively impacts the level of peripheral BDNF and decreases the level of inflammatory cytokines (McQuaid et al., 2013), thus enabling its normalization when pathologically altered (Dandi et al., 2018), which also leads to a significant improvement in hippocampal-related memory (Wang et al., 2019). EE also appears to normalize the level of estrogen and cortisol (Moreira et al., 2007). EE positively impacts the ability of estrogen to enhance spatial and object memory (Gresack et al., 2007). EE and estradiol have beneficial effects on hippocampal neurogenesis, and consequent memory performances. Sager et al. (2018) hypothesized that EE and estrogens might have a similar role in hippocampal neurogenesis improving cell proliferation in such regions, and thus benefiting memory performances.

EE increases the functional connectivity between the prefrontal cortex and hippocampus, and has enhanced learning and memory processes in rats with diencephalic amnesia (Ulrich et al., 2019). EE reduced the negative impact of stress on the spine density of basolateral amygdala neurons, thus promoting emotional regulation (Hegde et al., 2020). Also, EE reverses the negative behavioral and cardiovascular consequences associated with socially distressing situations promoting a sympathovagal balance (Normann et al., 2018).

Lastly, EE that includes social interactions leads to a better and faster recovery, rebalancing glucocorticoid secretion and inducing more remarkable neural plasticity after exposure to acute stress (Sharp et al., 2014).

## Enriched Interventions: A Holistic Therapeutic Proposal to Improve Survivors’ Quality of Life

IPV survivors are chronically exposed to physical, mental, and environmental stress due to recurrent even if unpredictable events accumulating over their lifespan (McEwen and Wingfield, 2003; Juster et al., 2010). From a biopsychosocial perspective, any environmental and social intervention could enhance survivors’ quality of life (Huefner et al., 2007). This has been demonstrated by translational model studying animals placed in socially and environmentally enriched places after long-term or acute stress.

IPV survivors may be exposed to severe forms of social isolation due to extremely controlling perpetrators. A supportive housing intervention is thus needed for the provision of rehabilitation therapies. Supportive housing can also act as a multi-modal rehabilitation program which rapidly enhances the survivors’ quality of life thanks to an EE based on new community and social connections (Figure 1B).

A recent systematic review (Yakubovich et al., 2022) highlighted the therapeutic potential of supportive housing for IPV survivors, in which a decrease in the intensity of psychological symptoms (depression, psychoactive addiction, anxiety, PTSD), alongside other mental health outcomes that increase the quality of life (e.g., intent to leave a partner, perceived safety, and housing and partner-related stress), has been shown amongst IPV survivors.

Despite the environmental and social support, O’Campo et al. (2016) raise questions regarding studies on the housing of IPV survivors since most focus on managing the financial and material difficulties as opposed to emotional ones: “mitigating psychological instability—issues of safety, promoting feelings of home, ensuring that new housing is a refuge—is often not considered when designing services for survivors of violence” (O’Campo et al., 2016, p 15).

In fact, safe, accessible, and affordable housing is essential to IPV response strategies, however focusing on survivors’ emotional needs is crucial to managing abuse trauma. The key to improving survivors’ quality of life would be a holistic intervention in which activities spanning from psychotherapy to psychosocial rehabilitation are offered to help their trauma management in the context of supportive housing.

Psychotherapy is crucial for the health promotion of IPV survivors, by providing a safe context in which victims can process traumatic events. Given the complexity of trauma, evidence-based psychotherapy with proven efficacy and effectiveness (Berg, 2019) is recommended. Cognitive behavioral therapies (CBT) and interpersonal therapies that were specifically tailored to IPV survivors, such as trauma-focused therapy, help decreased posttraumatic, depressive, and anxiety symptoms (Crespo and Arinero, 2010; Crespo et al., 2021).

## Discussion

IPV represents a global health priority. Despite the burgeoning focus on primordial and primary prevention of IPV, the goal is still extremely challenging.

Here we have examined the psycho-physio-pathological consequences of IPV and argue that evaluating the psychological and physiological parameters during treatments might enhance secondary (to prevent further psychophysiological consequences) and tertiary (to reduce the psycho-physio-pathological consequences experienced by survivors with adequate interventions) approaches. Prevention measures need to be emotionally-centered instead of merely economical, which can be achieved only with the addition of structured interventions such as psychotherapy.

## Data Availability Statement

The original contributions presented in the study are included in the article, further inquiries can be directed to the corresponding author.

## Author Contributions

VC, AV, and DM participated in all phases of the work. JA, NP, and AG contributed in writing and reviewing the work. VC, JA, NP, and DM contributed in conceiving and reviewing the work. All authors contributed to the article and approved the submitted version.

## Funding

MUR Ministero dell’Università e della Ricerca; (Italian Ministry of University and Research) and the University of Pisa funded the VC PhD scholarship.

## Conflict of Interest

The authors declare that the research was conducted in the absence of any commercial or financial relationships that could be construed as a potential conflict of interest.

## Publisher’s Note

All claims expressed in this article are solely those of the authors and do not necessarily represent those of their affiliated organizations, or those of the publisher, the editors and the reviewers. Any product that may be evaluated in this article, or claim that may be made by its manufacturer, is not guaranteed or endorsed by the publisher.
